# RelB^+^ Steady-State Migratory Dendritic Cells Control the Peripheral Pool of the Natural Foxp3^+^ Regulatory T Cells

**DOI:** 10.3389/fimmu.2017.00726

**Published:** 2017-06-22

**Authors:** Anja Döhler, Theresa Schneider, Ina Eckert, Eliana Ribechini, Nico Andreas, Marc Riemann, Boris Reizis, Falk Weih, Manfred B. Lutz

**Affiliations:** ^1^Institute for Virology and Immunobiology, University of Würzburg, Würzburg, Germany; ^2^Leibniz Institute on Aging – Fritz Lipmann Institute, Jena, Germany; ^3^Department of Pathology, Department of Medicine, NYU Langone Medical Center, New York, NY, United States

**Keywords:** dendritic cells, RelB, regulatory T cells, IL-2, lymph nodes

## Abstract

Thymus-derived natural Foxp3^+^ CD4^+^ regulatory T cells (nTregs) play a key role in maintaining immune tolerance and preventing autoimmune disease. Several studies indicate that dendritic cells (DCs) are critically involved in the maintenance and proliferation of nTregs. However, the mechanisms how DCs manage to keep the peripheral pool at constant levels remain poorly understood. Here, we describe that the NF-κB/Rel family transcription factor RelB controls the frequencies of steady-state migratory DCs (ssmDCs) in peripheral lymph nodes and their numbers control peripheral nTreg homeostasis. DC-specific RelB depletion was investigated in CD11c-Cre × RelB^fl/fl^ mice (RelB^DCko^), which showed normal frequencies of resident DCs in lymph nodes and spleen while the subsets of CD103^−^ Langerin^−^ dermal DCs (dDCs) and Langerhans cells but not CD103^+^ Langerin^+^ dDC of the ssmDCs in skin-draining lymph nodes were increased. Enhanced frequencies and proliferation rates were also observed for nTregs and a small population of CD4^+^ CD44^high^ CD25^low^ memory-like T cells (Tml). Interestingly, only the Tml but not DCs showed an increase in IL-2-producing capacity in lymph nodes of RelB^DCko^ mice. Blocking of IL-2 *in vivo* reduced the frequency of nTregs but increased the Tml frequencies, followed by a recovery of nTregs. Taken together, by employing RelB^DCko^ mice with increased frequencies of ssmDCs our data indicate a critical role for specific ssmDC subsets for the peripheral nTreg and IL-2^+^ Tml frequencies during homeostasis.

## Introduction

Dendritic cells (DCs) play roles not only in pathogen defense but also for maintaining tolerance to self-antigens (1–3). Immature DCs (iDCs) have been shown to exert their tolerogenic properties by inducing T cell anergy or regulatory T cells (Tregs) *in vitro* (4, 5). *In vivo* tolerance may be induced by lymph node resident iDCs that capture soluble antigens within the lymph node from the reticular conduit system (6). However, to promote antigen-specific tolerance against peripheral tissues, DCs must capture self-antigens in peripheral tissues and transport them into skin-draining lymph nodes for tolerogenic presentation to T cells (7). In fact, steady-state migratory DCs (ssmDCs) have shown a superior capacity to induce Treg *in vivo* as compared to immature resident subsets (8). DC migration in the steady state is accompanied by a partial maturation process (semimature) that is distinct from pathogen-matured DC by the lack of pro-inflammatory cytokine production (9). This concept has recently been confirmed by transcriptional profiling (10). Migration of ssmDCs into skin-draining lymph node T cell areas requires CCR7 expression (11). In the skin, ssmDCs can pick up soluble peptide antigens delivered by osmotic minipumps and the subsequent antigen presentation in the lymph nodes results in *de novo* conversion of naive T cells into induced Foxp3^+^ Tregs (iTregs) (12, 13). Using a murine transgenic model expressing OVA as a neo-self-antigen in the epidermis (K5-mOVA) we showed that also tissue-associated antigens are carried by ssmDCs, which are then cross-presented resulting in CD8^+^ T cell deletion (14) or *de novo* conversion of naive CD4^+^ T cells into Foxp3^+^ Tregs (15). In addition, we could show that the alternative NF-κB signaling pathway through RelB/p52 in regulating the function of Langerin^+^ dermal DCs (dDC) subset among the ssmDCs was critically involved in iTreg conversion (15). The functional importance of Treg induction by ssmDCs has been shown by their role in the protection from autoimmunity (8).

Members of the NF-κB family such as RelA, RelB, and c-Rel have mostly been associated with inflammation or immunogenicity for many cell types (16) including DCs (17). However, the functional role of this molecule in tolerogenic DCs is not fully understood since homozygous RelB^−/−^ mice lack peripheral lymph nodes (18), which do not allow us to study ssmDCs, and the results we obtained from heterozygous RelB^+/−^ (15) mice may involve indirect effects from other cell types. Therefore, we investigated the role of RelB in ssmDCs in more detail and the consequences for Treg induction. To address this we used mice expressing the Cre-recombinase under the murine CD11c promoter (CD11c-Cre mice) (19), which were crossed with mice where both alleles of the RelB gene were flanked by lox P sites (RelB^fl/fl^ mice). The resulting RelB^DCko^ mice allowed deeper molecular insights in Treg generation and maintenance in peripheral lymph nodes.

## Materials and Methods

### Mice

CD11cCRE mice express the Cre-recombinase under the CD11c promoter (19). In RelB^fl/fl^ mice, the exon 4 of the *relB* gene is flanked by loxP sites (20). RelB^fl/fl^ mice were mated with CD11cCRE mice to investigate the effects of a conditional deletion of RelB in DCs. Resulting CD11cCRE × RelB^fl/fl^ (subsequently referred to as RelB^DCko^) mice and corresponding control mice (RelB^fl/fl^ or CD11cCRE) were used at 5–12 weeks of age, if not otherwise indicated. C57BL/6 mice were purchased from Charles River (Sulzfeld, Germany) and bred in our respective facilities. OT-II mice were kindly provided by Francis Carbone, Melbourne, Australia and were crossed for some experiments with RAG1^−/−^ mice (gift from Thomas Winkler, University of Erlangen, Germany). Animal care, housing, and all experiments were performed according to institutional guidelines by the Animal Ethics Committee of the local authorities in Würzburg, Jena and New York, with age- and sex-matched animals.

### Cell Preparation

Skin-draining lymph nodes (inguinal, axillary, brachial, and popliteal), spleens, and thymi were cut into small pieces by using forceps and digested for 20 min at RT with 1 mg/ml DNase I (Roche) and 1 mg/ml collagenase III (Worthington) in RPMI 1640 medium supplemented with 10% FCS, 2 mM l-glutamine (PAA), 100 U/ml penicillin (PAA), and 100 µg/ml streptomycin (PAA). To disrupt multicellular complexes that include DC and T cells, 0.01 mM EDTA (Sigma) was added and incubation at RT was extended for 5 min. Cells were resuspended in ice cold PBS containing 5% FCS and 1 mM EDTA, transferred through a 70 µm cell strainer (BD) and counted. Splenic cells were additionally subjected to erythrocyte lysis before enzymatic digestion. BM-DC were prepared as described in detail before (21).

### Antibodies and Flow Cytometry

For cell surface staining the following monoclonal antibodies (mAbs) and conjugates from Biolegend were used: B220 (clone RA3-6B2), CD4 (clone RM4-5 and GK1.5), CD8α (clone 53-6.7), CD11c (clone N418), CD25 (clone PC61 and 7D4), CD40 (clone 3/23), CD80 (clone 16-10A1), CD86 (clone GL-1), CD103 (clone M290), GITR (clone DTA-1), MHC II (clone 2G9 and M5/114.15.2), PD-1 (clone J43), and PDCA-1 (clone 927). Antibodies for CD44 (clone IM7) and Vβ5.1/5.2 (clone MR9-4) were purchased from BD. If primary antibodies were biotinylated, the following secondary antibodies were used: streptavidin–FITC, –PE, –PECy7 (Biolegend), or streptavidin–Pacific Blue (Invitrogen).

Intracellular staining of Foxp3 (clone FJK-16s, eBiosciences), Helios (clone 22F4, Biolegend), and Ki67 (clone B56, BD) was performed in accordance with the manufacturer’s protocol of the Anti-Mouse Foxp3 Staining Set (eBiosciences). Flow cytometric staining of RelB was done by using first a polyclonal rabbit anti-mouse RelB antibody (C-19, Santa Cruz) followed by a goat anti-rabbit-DyLight488 antibody (Jackson) as described previously (15). For detection of intracellular Langerin, cells were fixed and permeabilized in Cytofix/Cytoperm (BD) for 20 min at RT, washed in Perm/Wash Buffer (BD), and incubated with a rat anti-mouse Langerin-AlexaFluor488 (clone 929F3.01, Dendritics) for 30 min at 4°C.

All flow cytometric analyses were performed on a FACSCalibur, FACSCanto II, or LSRII (BD) and data were analyzed by FlowJo Software (Tree Star).

### *Ex Vivo* Stimulation and Cytokine Staining of Cells from Lymph Nodes

Single cell suspensions from skin-draining lymph nodes were prepared as described above and resuspended at a density of 4 × 10^6^ cells per ml in complete RPMI 1640 medium. Cells were then stimulated with 10 ng/ml PMA (Sigma) and 1 µg/ml ionomycin (Sigma) in the presence of 5 µg/ml brefeldin A (Sigma) for 5 h at 37°C. After stimulation, cells were stained for surface markers (CD4, CD11c, CD25, and CD44), fixed in 2% formaldehyde for 20 min at RT, and stained intracellularly with an IL-2 specific antibody (clone JES6-5H4, Biolegend) in Perm Buffer (PBS supplemented with 0.5% saponin, 0.1% BSA, and 0.05% sodium azide) for 30 min at 4°C. Control straining was performed with a PE-conjugated rat IgG2b isotype.

### Implantation of Micro-osmotic Pumps Secreting OVA_327-339_ Peptide

Micro-osmotic pumps (ALZET, model 1002, Charles River) were filled with PBS or OVA_327-339_ peptide, respectively, to deliver 10 µg antigen per day as described previously (12). To decrease the start-up time for continuous and reliable pumping, prefilled micro-osmotic pumps were placed in PBS for at least 6 h at 37°C before implantation. For subcutaneous implantation, a small incision was made on the back next to the hips, a subcutaneous pocket was formed, and the pump was inserted. The wound was closed by saturation and using AutoClip Wound Closure System (Charles River). Adoptive transfer of T cell was carried out 1 day after implantation.

### Adoptive Transfer of T Cells from WT and OT-II Mice

Single cell suspensions of lymph nodes (pooled skin-draining and mesenteric lymph nodes) and spleens from WT, OT-II, and OT-II.Rag1^−/−^ mice were prepared as described above. CD4^+^ T cells were purified by negative magnetic separation using either the Mouse CD4 T Cell Enrichment Kit (StemCell) or the Mouse CD4^+^ T Cell Isolation Kit II (Miltenyi Biotec) according to the manufacturer’s instructions. For further isolation of CD25^+^ CD4^+^ T cells from WT and OT-II mice a positive magnetic separation was performed using Mouse CD25 Micro Beads (Miltenyi Biotec) in accordance to the recommendations of the manufacturer. The purity of the isolated T cell populations was assessed by flow cytometry (CD4^+^ > 90%, CD4^+^ CD25^+^ > 60%). For labeling with carboxyfluorescein diacetate succinimidyl ester (CFSE) cells were resuspended at a density of 1 × 10^7^ cells per ml in PBS containing 2.5 µM CFSE (Molecular Probes, Invitrogen) for 15 min at RT. Subsequently, cells were washed with complete RPMI 1640 medium, resuspended in an appropriate volume of PBS, and injected into the lateral tail vein of recipient mice. After the indicated time points recipient mice were sacrificed and cell suspensions from skin-draining lymph nodes and spleens were prepared. Cells were stained with antibodies for CD4 and Foxp3 and analyzed by flow cytometry.

### Treatment with Anti-IL-2 Antibody

To neutralize IL-2, mice received intraperitoneally 2 mg anti-IL-2 mAb (clone S4B6, BioXCell). Before injection and at the indicated time points after injection blood was taken from submandibular vein, stained for CD4, Foxp3, and Ki67, and analyzed by flow cytometry.

### RNA Isolation and qRT-PCR

Total RNA was isolated from sorted T cells and DC using Micro Rneasy Kit (Qiagen) according to manufacturer’s instructions. mRNA was amplified (Nano or Pico AmpTec Express Art Kit), digested with DNAse I (MBI Fermentas), and reverse transcribed using iScript (Biorad) in accordance to the recommendations of the manufacturer. Quantitative expression analysis of IL-2, IL-7, IL-15, and RelB was determined with a iCycler iQ (Biorad) using the following primers: mIL-2-FOR TTTGAGTGCCAATTCGATGA, mIL-2-REV AGGGCTTGTTGAGATGATGC, mIL-7-FOR2 TCAGCATCGATGAATTGGAC, mIL-7-REV2 CCAGTGTTTGTGTGCCTTGT, mIL-15-FOR CATTTTGGGCTGTGTCAGTG, mIL-15-REV TGCAACTGGGATGAAAGTCA, RT-RelB-F CCGAGCTAGGGGCCTTGGGTTCC, and RT-RelB-R AGCTCGATGGCGGGCAGGGTCTTG. qRT-PCR was performed in 25 µl SYBR Green Master mix (MBI Fermentas) containing 1 µl cDNA (undiluted or 1:10 diluted) and 0.2 µM of forward and reverse primers. The PCR program was: 95°C for 14 min and then 40 cycles of 95°C for 30 s, 60°C for 30 s, and 72°C for 30 s followed by a melting curve analysis. Relative quantification of the samples was performed by the comparative ΔΔ cycle threshold method. For normalization of the samples the housekeeping gene β-actin (β-Actin-F QRT CATTGCTGACAGGATGCAGA, β-Actin-R QRT TTGCTGATCCACATCTGCTG) was used.

### Immunofluorescence and Confocal Microscopy

To detect and calculate DC in skin, immunofluorescence stainings of MHC II^+^ cells were performed using either epidermal sheets or frozen cryostat sections of ear skin. Epidermal sheets were prepared as described previously (22). Briefly, ears were washed in 70% ethanol and dried at RT. Ventral and dorsal sides of each ear were separated with fine forceps and the sides with dermis down were floated on 0.5 M ammonium thiocyanate for 25 min at 37°C. Sides were washed with PBS, and the epidermis was peeled off the dermis, cut into small pieces, and fixed for 20 min with acetone at RT. After several washing steps (2× PBS, 2× 1% BSA/PBS), epidermal sheets were incubated with 10% BSA/PBS for 30 min to block unspecific binding of antibodies, stained with a pure rat anti-mouse MHC II (clone 2G9, BD) or a rat IgG2a isotype (BD) overnight at 4°C followed by a donkey anti-rat IgG F(ab′)2-AlexaFluor488 (Jackson) for 30 min at RT. Immunolabeled epidermal sheets were mounted in Vectashield Mounting Medium (Vector Laboratories) and viewed on a fluorescence microscope (DM IRE220, Leica). Skin cryostat sections (9 µm) were fixed with ice cold acetone for 15 min, dried for 30 min, and rehydrated in PBS followed by blocking in 10% BSA/PBS for 30 min at RT. Immunofluorescence staining and microscopically imaging of MHC II^+^ cells were performed as described for epidermal sheets.

### Statistics

Statistical analyses were performed using Prism 5.0 software (GraphPad Prism). The unpaired, two-tailed Student’s *t*-test was used, if data sets of two independent groups were normally distributed. The Mann–Whitney test was performed when data sets of two groups were not normally distributed.

## Results

### RelB^DCko^ Mice Show Increased ssmDC Frequencies in Peripheral LN (PLN)

**Figure 1:**
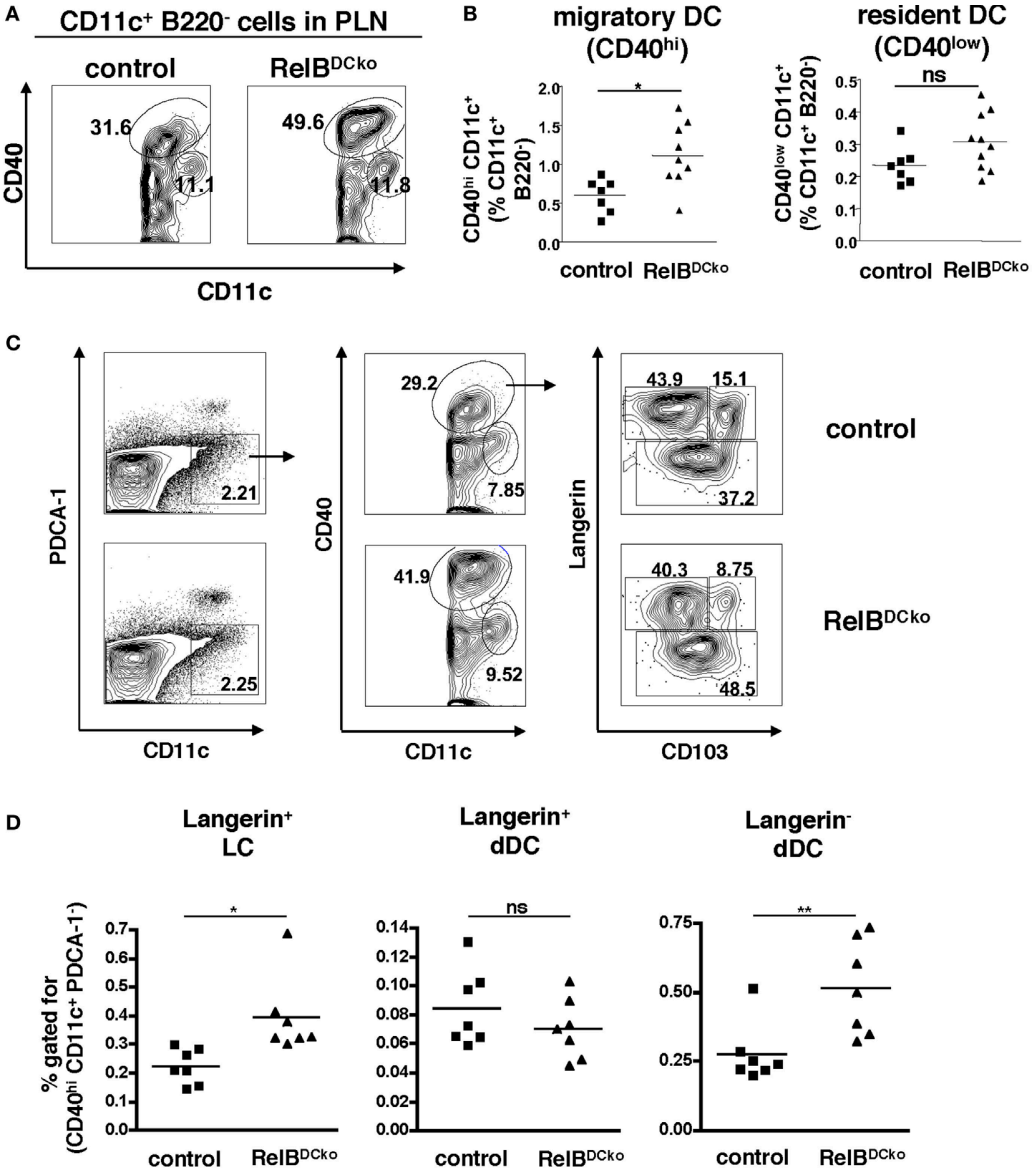
RelB^DCko^ mice show an increased frequency of Langerhans cells (LCs) and Langerin^−^ dermal DCs (dDCs) among steady-state migratory DCs in skin-draining lymph nodes. **(A,B)** Cells from peripheral LN (PLN) of control and RelB^DCko^ mice were stained for B220, CD11c, and CD40 and analyzed by flow cytometry. **(A)** Contour plots represent CD11c^+^ B220^−^ cells within a FSC/SSC gate for live cells. Based on the expression of CD40 migratory CD40^hi^ dendritic cell (DC) and resident CD40^low^ DC are distinguished among CD11c^+^ B220^−^ cells. B220^−^ CD11c^low^ CD40^low^ cells represent RelB^−^ macrophages. Numbers indicate the percentages of cells within the gate. **(B)** Statistical summary of frequencies of migratory and resident DC among CD11c^+^ B220^−^ cells PLN of RelB^DCko^ (*n* = 11) or control mice (*n* = 7). Percentages of both DC subsets among CD11c^+^ B220^−^ cells were calculated in relation to the absolute frequency of CD11c^+^ B220^−^ cells. Each symbol represents an individual mouse and black lines indicate the mean values. Statistical analyses were performed using the Mann–Whitney Test: ns, not significant, *p < 0.05. **(C)** Representative flow cytometric analysis of migratory DC subpopulation in PLN of control and RelB^DCko^ mice after surface staining of CD11c, CD40, and CD103 and intracellular staining of Langerin. After gating specifically on CD11c^+^ B220^−^ cells (right panel), migratory CD40^hi^ DCs (middle panel) were further analyzed for their expression of Langerin versus CD103. Three migratory DC subsets could be defined: Langerin^+^ CD103^−^ LC, Langerin^+^ CD103^+^ dDC, and Langerin^−^ CD103^+/−^ dDC (left panel). Numbers indicate the percentages of cells within each gate. **(D)** Frequencies of Langerin^+^ CD103^−^ LC, Langerin^+^ CD103^+^ dDC, and Langerin^−^ CD103^+/−^ dDC among the total percentages of CD40^hi^ CD11c^+^ B220^−^ cells in PLN of control (*n* = 7) and RelB^DCko^ mice (*n* = 7) are shown. Data represent the mean values + SD from two independent experiments with 3–4 mice per genotype and each experiment. Statistical *p*-values were obtained by performing two-tailed unpaired Student’s *t*-test: ns, not significant, *p < 0.05, **p < 0.01.

Previously, we could show in RelB^+/−^ mice that ssmDCs of skin-draining PLN expressed the highest levels of RelB under steady-state conditions (15). Here, we tested whether DC-intrinsic deletion of RelB in RelB^DCko^ mice affected the ssmDC populations in PLNs. Intracellular FACS analysis of DCs from thymus, peripheral lymph nodes, and spleen confirmed highest RelB expression in PLNs and this could not be detected in RelB^DCko^ mice (Figures S1A,B in Supplementary Material), while similar levels of surface MHC II and CD80/CD86 costimulatory molecules were detected (Figures S2A,B in Supplementary Material). Further analyses of PLN DC indicated that the CD11c^+^ CD40^high^ ssmDCs but not the CD11c^+^ CD40^low^ resident DCs were increased in RelB^DCko^ mice (Figures 1A,B), while the total lymph node and spleen cell numbers remained unchanged (Figure S3 in Supplementary Material). In addition, also the skin distribution and frequency of DCs remained unaltered (Figure S4 in Supplementary Material). The ssmDCs in PLN homogenously express RelB (Figures S1C,D in Supplementary Material) and consist of three major subsets composed of Langerin^+^ CD103^−^ epidermal Langerhans cells (LCs), Langerin^+^ CD103^+^ dDCs, and Langerin^−^ CD103^−^ dDCs (Figure 1C) (23). When these ssmDCs were analyzed, we found increased proportions of LCs and Langerin^−^ dDCs, while Langerin^+^ dDCs remained unchanged in RelB^DCko^ mice (Figure 1D). Increased frequencies of DC subsets may result from enhanced proliferation or reduced apoptosis. However, ssmDCs did not show altered proliferation rates as detected by Ki67 staining (Figure S5A in Supplementary Material) or changes in Annexin V binding as a sign of apoptosis (Figure S5B in Supplementary Material). Thus, the reason for the selective increase in some but not all ssmDC subsets remains open.

### RelB^DCko^ Mice Show Increased Frequencies of Proliferating Tregs in PLNs

**Figure 2:**
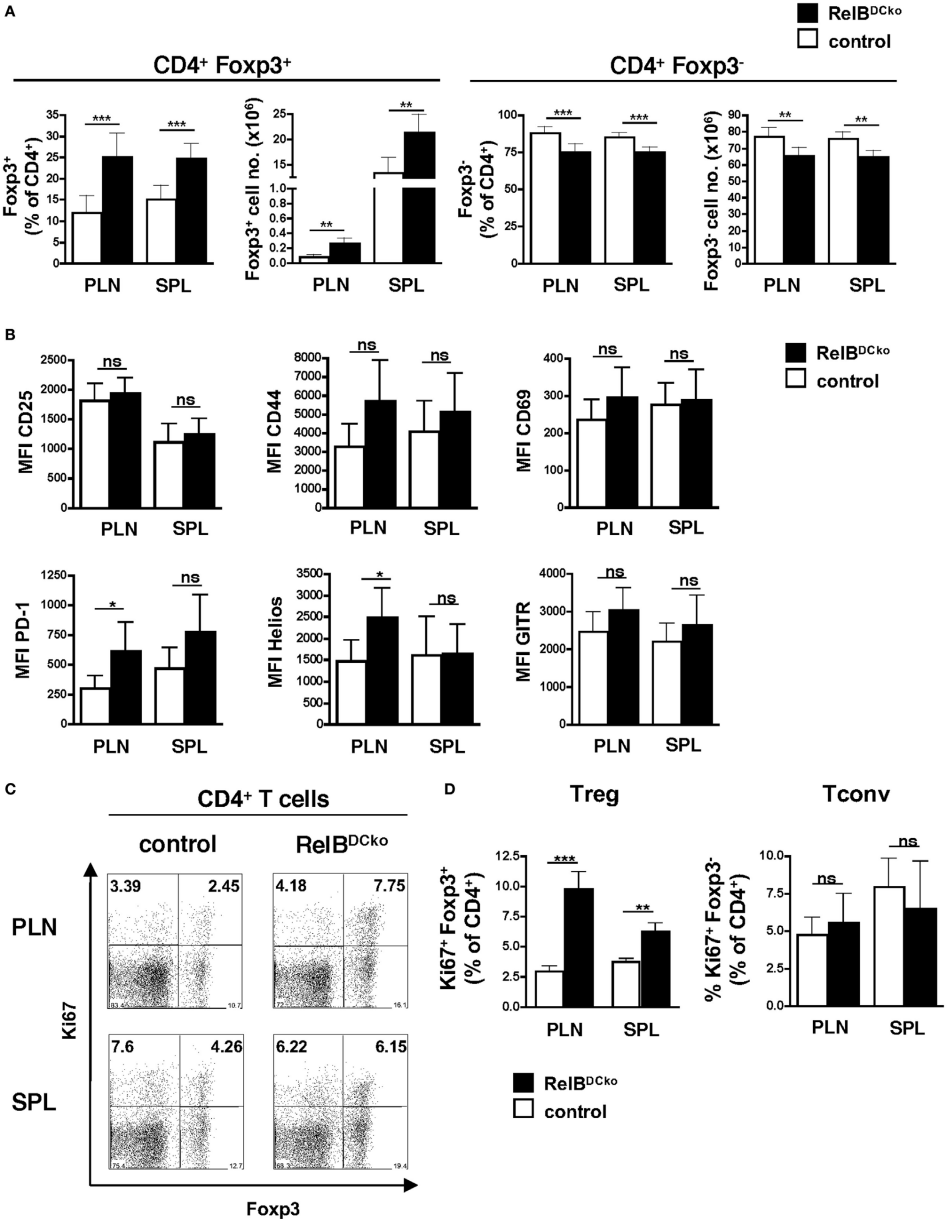
Mice with a RelB deficiency in dendritic cell display an increased frequency of proliferating Foxp3^+^ regulatory T cells (Tregs). **(A)** Cells from peripheral LN (PLN) and SPL of control and RelB^DCko^ mice were analyzed by flow cytometry for surface CD4 and intracellular Foxp3 expression. Percentages and absolute numbers of Foxp3^+^ Treg and Foxp3^−^ conventional T cells (Tconv) among CD4^+^ T cells in peripheral lymphoid organs of control mice (*n* ≥ 7) and RelB^DCko^ mice (*n* = 7) are shown. **(B)** Geometric MFI of CD25, CD44, CD69, PD-1, Helios, and GITR expression by CD4^+^ Foxp3^+^ Treg in PLN and SPL of control mice (*n* ≥ 3) and RelB^DCko^ mice (*n* ≥ 3) are displayed. **(C,D)** Cells from PLN and SPL of control and RelB^DCko^ mice were analyzed by flow cytometry for surface CD4 and intracellular Foxp3 and Ki67 expression. **(C)** Representative dot plots showing Ki67 and Foxp3 expression among CD4^+^ T cells. Numbers indicate the percentages of cells in each quadrant. **(D)** Percentages of proliferating Ki67^+^ Foxp3^+^ Treg and Ki67^+^ Foxp3^−^ Tconv among CD4^+^ T cells in PLN and SPL of control mice (*n* = 8) and RelB^DCko^ mice (*n* = 6) are shown. **(A,B,D)** Data represent the mean values + SD. Mann–Whitney test was used for statistical analyses: ns, not significant, *p < 0.05, **p < 0.01, ***p < 0.001.

Since we showed before that Langerin^+^ dDCs were the decisive subset of ssmDCs in PLNs that converted naive CD4^+^ T cells into Foxp3^+^ Tregs (15), we analyzed the Treg population of RelB^DCko^ mice. Frequencies and absolute numbers of CD4^+^ Foxp3^+^ Tregs were increased in PLNs of RelB^DCko^ mice, while CD4^+^ Foxp3^−^ conventional T cells (Tconv) were decreased (Figure 2A). This was not observed for total CD4^+^ or CD8^+^ T cells or B cells in PLNs or spleen (Figure S6 in Supplementary Material). The CD4^+^ Foxp3^+^ Tregs of RelB^DCko^ mice showed an activated phenotype by expressing significantly higher levels of PD-1 and Helios, while no or only trends of higher CD25, CD69, CD44, and GITR surface expression levels could be observed (Figure 2B). In addition, these activated Tregs also showed higher proliferation rates as indicated by elevated Ki67 expression (Figures 2C,D).

In order to test whether increased Treg frequencies could be a result of enhanced thymic output, the thymocyte frequencies of these mice were analyzed. The data indicate that already in the thymus CD4^+^ CD8^−^ Foxp3^+^ T cells appeared at a higher frequency and were proliferating whereas other thymocyte populations remained normal (Figures S7A–E in Supplementary Material). These data indicate that the frequency of proliferating nTregs is increased in thymus and PLNs of RelB^DCko^ mice.

### Decreased iTreg Conversion Potential but Expansion of nTregs in RelB^DCko^ Mice

**Figure 3:**
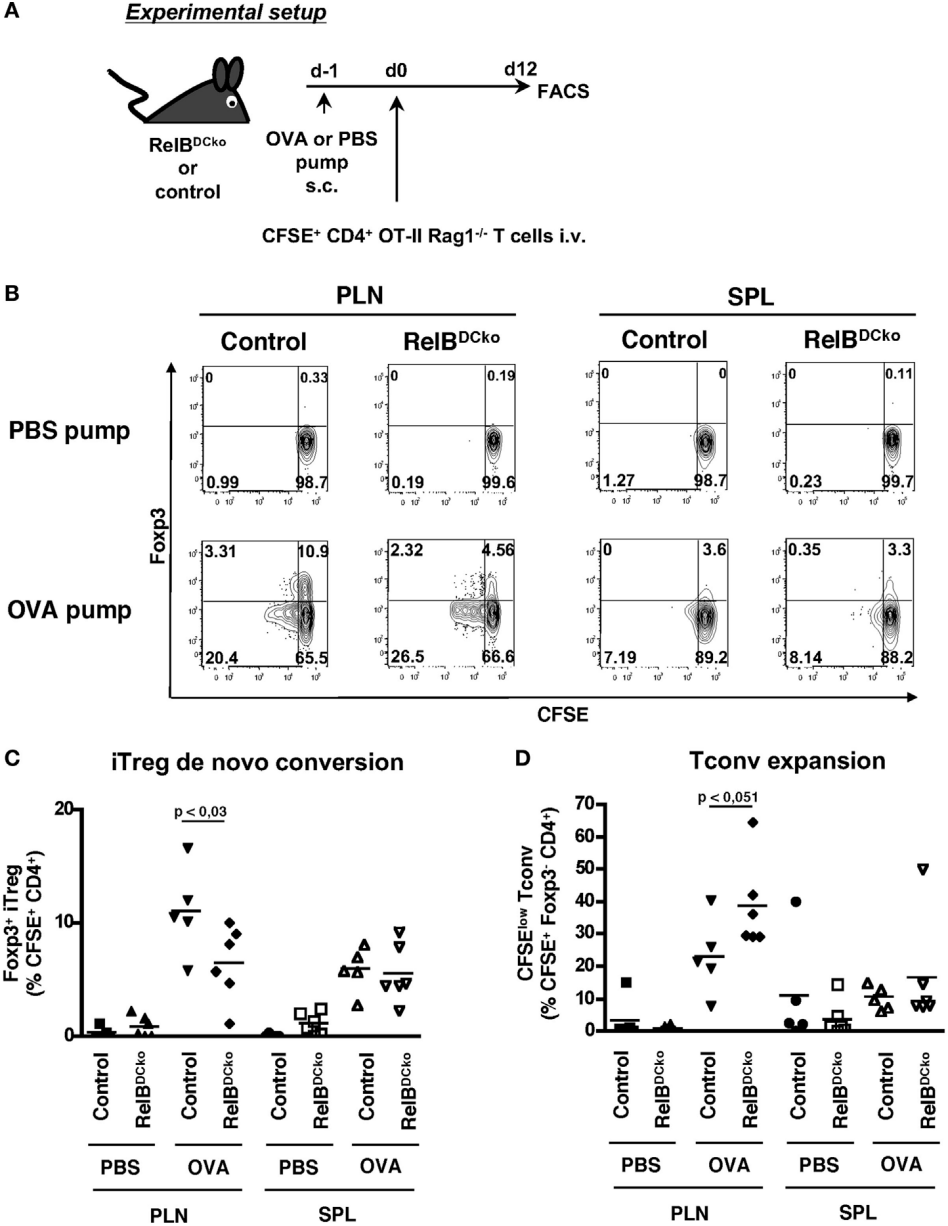
RelB^DCko^ mice do not promote enhanced induction of iTregs. **(A)** Scheme of experimental setup. Micro-osmotic pumps loaded with PBS or OVA peptide were subcutaneously implanted in RelB^DCko^ and control mice. One day later mice received an intravenous injection of 4.5–5 × 10^6^ carboxyfluorescein diacetate succinimidyl ester (CFSE)-labeled CD4^+^ CD25^−^ OT-II × Rag1^−/−^ T cells. Twelve days after adoptive transfer, cell suspensions from peripheral LN (PLN) and SPL of RelB^DCko^ and control mice were stained for CD4 and Foxp3 and analyzed by flow cytometry. **(B)** Representative dot plots showing CFSE and Foxp3 expressing among transferred CFSE^+^ CD4^+^ T cells in PLN and SPL. Numbers indicate percentages of cells in each quadrant. **(C)** Percentages of Foxp3^+^ iTregs among CFSE^+^ CD4^+^ T cells in PLN and SPL of RelB^DCko^ (*n* ≥ 6) and control mice (*n* ≥ 5) are depicted. **(D)** Frequencies of proliferating CFSE^low^ Foxp3^−^ conventional T cells among CFSE^+^ CD4^+^ Foxp3^−^ T cells in PLN and SPL of RelB^DCko^ (*n* ≥ 6) and control mice (*n* ≥ 5). **(B–D)** Data represent the mean values + SD from three independent experiments with *n* ≥ 2 mice per genotype and condition. Statistical analyses were performed using Mann–Whitney test: ns, not significant, *p* < 0.05.

Although the results may point to the thymic output as the responsible mechanism for a higher Treg frequency observed in spleen and lymph nodes, these data cannot explain the continuation of proliferation of Tregs in the PLN and spleen. Furthermore, it remained unclear whether the Treg increase was due to enhanced iTreg generation or expansion of nTregs and whether the effects depend on self-antigen. To clarify these points adoptive transfer experiments were performed using T cells from naive CD4^+^ OT-II × RAG1^−/−^ mice. These mice lack both the endogenous repertoire of T cells and CD4^+^ Foxp3^+^ transgenic OT-II Tregs. Thus, the transferred cells constituted exclusively of naive CD4^+^ Foxp3^−^ transgenic OT-II T cells and the appearance of CD4^+^ Foxp3^+^ transgenic OT-II Tregs cells would indicate *de novo* conversion of naive CD4^+^ OT-II T cells into iTregs. After transfer of naive CD4+ OT-II × RAG1^−/−^ T cells into control or RelBDCko mice OVA-loaded osmotic minipumps were implanted under the skin as a source of soluble peripheral self-antigen (15). The results showed a lower antigen-specific iTreg conversion into CD4^+^ Foxp3^+^ Tregs (Figures 3A,B) and Tconv a trend for an increased proliferation rate in RelB^DCko^ mice (Figure 3C). Importantly, *de novo* conversion of naive T cells into iTregs is not increased in RelB^DCko^ mice.

**Figure 4:**
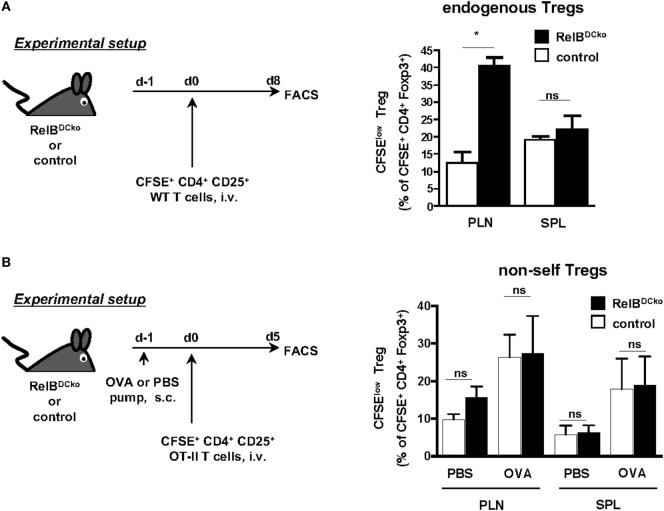
Expansion of self-antigen-specific Foxp3^+^ nTregs in peripheral lymph nodes of RelB^DCko^ mice. **(A)** Carboxyfluorescein diacetate succinimidyl ester (CFSE)-labeled CD4^+^ CD25^+^ T cells (3 × 10^6^) from wild-type mice were intravenously transferred into RelB^DCko^ and control mice. Eight days later cell suspensions from peripheral LN and SPL were stained for CD4 and Foxp3 and analyzed by flow cytometry. Graphs display the percentages of proliferating (CFSE^low^) cells among CFSE^+^ CD4^+^ Foxp3^+^ T cells (right) as mean value + SD. Data shown are representative of two independent experiments with one mouse per genotype in each experiment. **(B)** PBS- or OVA peptide-loaded osmotic minipumps were subcutaneously implanted in RelB^DCko^ and control mice. One day later mice received an intravenous injection of 1.4 × 10^6^ CFSE-labeled CD4^+^ CD25^+^ OT-II T cells. For flow cytometric analysis, mice were sacrificed 5 days after adoptive transfer and stained for CD4 and Foxp3. Graphs display the percentages of proliferating CFSE^low^ cells among all transferred CFSE^+^ CD4^+^ Foxp3^+^ nTregs. Data represent the mean values + SD from one representative experiment with *n* = 2–5 mice per genotype and condition. **(A,B)** Statistical analyses were performed using unpaired student’s *t*-test: ns, not significant, *p < 0.05.

Conversely, adoptively transferred thymus-derived nTregs can be tested for their antigen-dependent expansion in peripheral lymph nodes. To distinguish whether only self-antigen-specific nTreg can be stimulated in lymph nodes or also nTreg with foreign antigen specificity we used two different settings of nTreg transfer. In naive Wt mice, the CD4^+^ CD25^+^ nTregs are predominantly specific for self-antigens (24). After transfer of self-antigen-specific nTregs we detected their enhanced proliferation in PLN of RelB^DCko^ but not control mice or the spleen (Figure 4A). Since this increased nTreg proliferation correlated with increased frequencies of self-antigen transporting LCs and CD103^−^ Langerin^−^ dDCs we hypothesized that antigen presentation of these two DC subsets may play a role.

In the second setting, we transferred nTregs from OT-II mice that are specific for foreign OVA antigen and express a high affinity TCR, since OVA is not expressed in the thymus of OT-II mice. Then PBS- or OVA-loaded minipumps as a source of antigen were provided. Here, an increased expansion of CD4^+^ CD25^+^ nTregs from OT-II mice could be observed in mice carrying OVA-pumps, indicating a supporting role of antigen for nTreg proliferation (Figure 4B). However, a higher expansion rate of nTregs in RelB^DCko^ mice than in control mice could not be observed after the implantation of OVA-loaded minipumps (Figure 4B). The results above (Figure 3) and our previous findings showed that the Langerin^+^ dDCs were responsible for OVA transport and presentation as well as *de novo* conversion of iTregs (15). These results are consistent with the fact that in PLNs of RelB^DCko^ mice with the frequency of CD103^+^ Langerin^+^ dDC was not increased also the iTreg conversion rate was not enhanced. In contrast, increased frequencies of CD103^−^ Langerin^−^ dDCs and LCs in PLNs of RelB^DCko^ mice correlated with an enhanced proliferation rate of nTreg. These data may indicate a division of labor among ssmDC subsets from the skin for iTreg conversion by CD103^+^ Langerin^+^ dDCs and nTreg stimulation by CD103^−^ Langerin^−^ dDCs and/or LCs. Although the role of LCs cannot be clearly deducted from our experiments, others have shown that resting LCs in the epidermis stimulate skin resident Treg activation and proliferation (25) and that glucocorticoids further promote TGF-β production by LCs to enhance Treg numbers in contact hypersensitivity patients (26).

Together, these data obtained from RelB^DCko^ mice indicate that beyond the thymic selection also self-antigen presentation by Langerin^−^ dDCs and/or LCs in PLNs regulates the pool of self-antigen-specific nTregs.

### CD4^+^ CD44^high^ CD25^low^ Memory-Like T Cells (Tml) Show Increased IL-2 Production in Steady-State PLNs of RelB^DCko^ Mice

**Figure 5:**
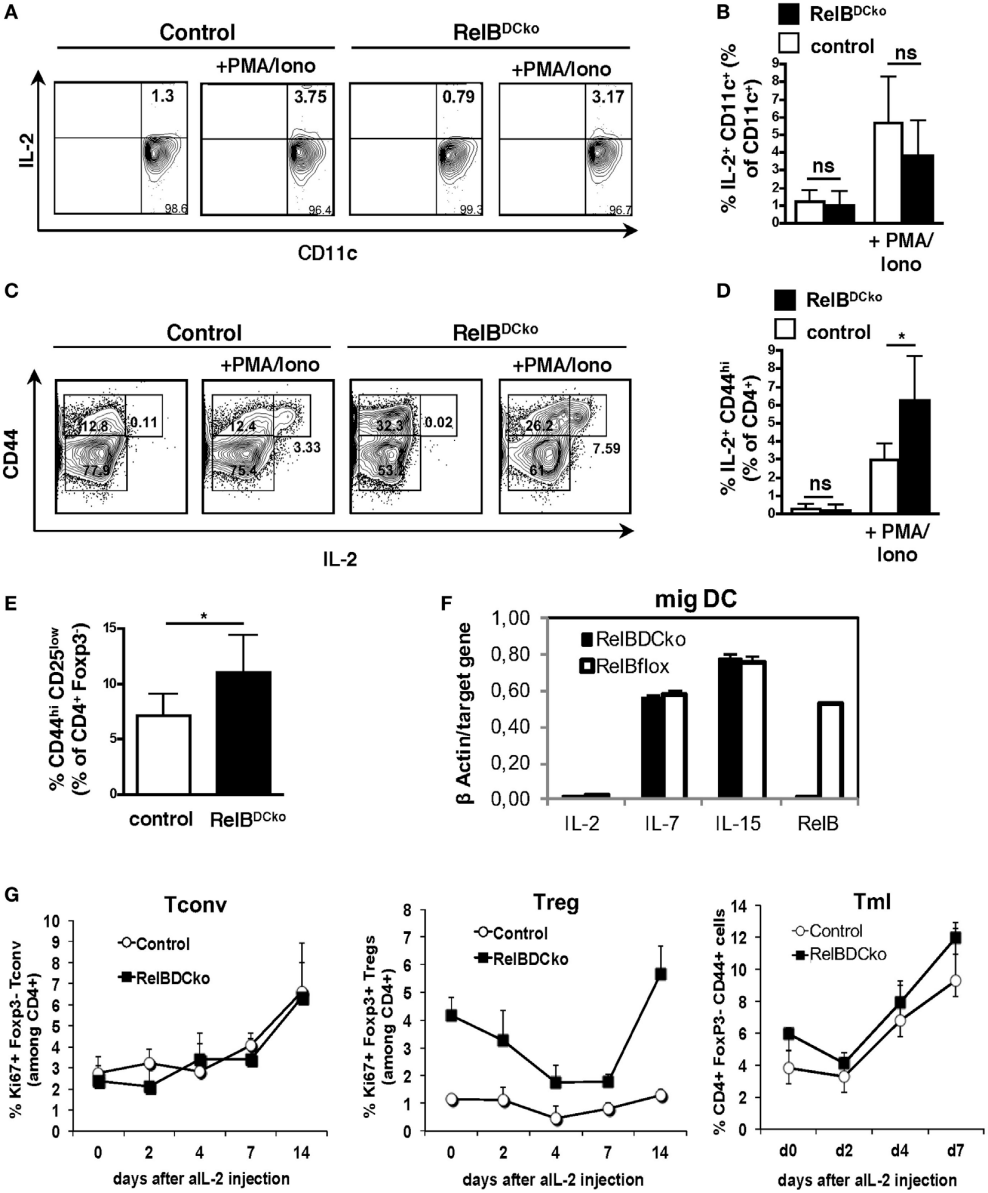
IL-2 produced by CD4^+^ CD25^int^ CD44^hi^ T cells is required for regulatory T cell (Treg) maintenance in RelB^DCko^ mice. **(A–D)** Single cell suspensions from peripheral LN (PLN) of control and RelB^DCko^ mice were either left unstimulated or activated with PMA/ionomycin for 5 h in the presence of brefeldin A. After incubation cells were stained (surface: CD11c, CD4, and CD44; intracellular: IL-2) and analyzed by flow cytometry. **(A)** Representative dot plots showing expression of CD11c and IL-2 among CD11c^+^ dendritic cell (DC). Numbers in each quadrant indicate the percentages of cells gated on CD11c^+^ cells. **(B)** Graphs display the percentages of IL-2^+^ CD11c^+^ DC among CD11c^+^ DC compiled from five independent experiments with one mouse per genotype and condition in each experiment. **(C)** Representative dot plots showing expression of CD44 and IL-2 among CD4^+^ T cells. Numbers in the quadrants indicate the percentages of cells gated on CD4^+^ cells. **(D)** Compiled date from percentages of IL-2^+^ CD44^+^ cells among CD4^+^ T cells of five independent experiments with one mouse per genotype and condition in each experiment are displayed. **(E)** Percentages of CD25^int^ CD44^hi^ cells among CD4^+^ T cells in PLN of control (*n* = 6) and RelB^DCko^ mice (*n* = 7) from five independent experiments are shown. **(F)** mRNA expression of IL-2, IL-7, and IL-15 in sorted migratory CD40^hi^ DC from PLN of control and RelB^DCko^ mice analyzed by qRT-PCR. **(G)** Injection of an IL-2 neutralizing antibody abrogated Treg but not conventional T cells (Tconv) cell division in RelB^DCko^ mice. 1 mg αIL-2 antibody was injected intraperitoneal into RelB^DCko^ and control mice. Before injection and 2, 4, 7, 14, and 21 days afterward blood samples were taken, stained for CD4, CD44, Foxp3, and Ki67, and analyzed by flow cytometry. Kinetics of the percentages of proliferating CD4^+^ Ki67^+^ Foxp3^−^ Tconv, CD4^+^ Ki67^+^ Foxp3^+^ Tregs, and CD4^+^ CD44^high^ Foxp3^−^ memory-like T cells after αIL-2 injection are shown in the diagram. Three mice per genotype and experiment were used. Depicted is one representative experiment from four experiments. **(B,D,E,F)** Data represent the mean values + SD. For statistical analyses student’s *t*-test was performed: ns, not significant, *p < 0.05.

During the steady state, the niche size for nTreg in the thymus is controlled by the availability of IL-2 provided by DCs (27). Also, peripheral maintenance of nTregs is dependent on IL-2 (28, 29), which is secreted from presumably self-reactive CD4^+^ CD25^low^ non-Tregs (30). Here, we sought to identify the source of increased IL-2-competent cells in PLNs by real-time PCR and FACS analyses in RelB^DCko^ mice. Total PLN cells were stimulated with PMA + ionomycin and the only IL-2 producing cells were identified as CD11c^+^ DC (Figures 5A,B) and CD4^+^ CD25^low^ CD44^high^ Tml (Figures 5C,D). An increase in the frequency of IL-2 producing cells in RelB^DCko^ mice was observed only for the Tml but not the DCs (Figures 5A–D) together with the total frequency of CD4^+^ CD44^high^ T cells in PLNs of RelB^DCko^ mice (Figure 5E). These data suggest that IL-2^+^ Tml and not DCs may contribute to the elevated Treg proliferation in RelB^DCko^ mice. To substantiate the results, we sorted the ssmDC populations from PLN of RelB^DCko^ and control mice and tested their IL-2, IL-7, and IL-15 production at real-time PCR level. These data indicate that IL-2 is not produced by DCs in PLNs and no differences were found for the other cytokines between RelB^DCko^ and control mice (Figure 5F). To demonstrate that IL-2 is functionally required for Treg proliferation in the RelB^DCko^ mice we injected a blocking anti-IL-2 antibody (clone S4B6) and found that proliferating Ki67^+^ CD4^+^ Foxp3^+^ Treg but not Ki67^+^ CD4^+^ Foxp3^−^ Tconv frequencies in blood dropped with a maximum between day 4 and 7 after injection and recovered thereafter (Figure 5G). In contrast to Treg and Tconv, Tml frequencies increased at days 4 and 7. Thus, the CD4^+^ Tml population in RelB^DCko^ mice is not only increased in their cellular frequency but also the proportion of IL-2 producers among them is elevated as compared to control mice, thereby enabling higher nTreg proliferation and pool sizes in PLNs of RelB^DCko^ mice.

## Discussion

Here, we investigated the cellular composition of lymphoid organs in mice deficient for RelB expression in CD11c^+^ DCs under steady-state conditions, termed RelB^DCko^ mice. The results indicate an increased frequency of epidermal LCs and CD103^−^ Langerin^−^ dDCs in the PLNs of RelB^DCko^ mice as compared with control mice. We could not answer the question how RelB controls the frequency of these ssmDC subsets in RelB^DCko^ mice. Their distribution in the skin and their apoptosis rate in PLNs appeared normal. However, RelB could influence the expression of maturation markers and chemokine receptors differentially. After contact sensitivity responses it was found that LCs and dDCs display differences in activation markers and homing (31). The production of CCL17 by ssmDCs in lymph nodes is restricted to CD11^+^ CD8^−^ DC subsets (32) and its secretion further impacts migration patterns by other DCs by controlling their responsiveness to CCR7 ligands and CXCL12 (33). Thus, further analyses on the functional consequences of RelB deficiency in DCs are needed.

Further analysis of cellular subsets in PLNs of RelB^DCko^ mice indicated changes in the frequencies of specific T cell subsets. The increase of the two ssmDC subsets was accompanied by increased frequencies of CD4^+^ CD25^+^ Foxp3^+^ nTregs and CD4^+^ CD25^low^ CD44^high^ Tml. Tml have been shown before to represent the responsible T cell subset in the steady state that provides IL-2 for nTreg proliferation and maintenance, required to prevent autoimmunity (30). Tml represent a small proportion of CD4^+^ Tconv with low-affinity TCRs for self-antigen that escape the thymic negative selection (34). In the thymus, Tregs recognize IL-2 derived from DCs that seems to regulate the thymic Treg pool size (27). In the periphery, steady-state presentation of self-antigens together with IL-2 production has been demonstrated to be the critical components for nTreg survival (28). Treg and IL-2^+^ Tml locate in close proximity in lymph nodes (35). Here, we found that increased frequencies of ssmDCs are accompanied by increased proliferation rates of only two other populations, namely nTregs and IL-2^+^ Tml. DC-mediated self-antigen presentation may stimulate Tml to release IL-2 that supports nTreg steady-state proliferation and maintenance (35). The requirement for self-antigen presentation is supported by our findings that adoptively transferred self-antigen-specific WT nTregs but not foreign antigen-specific OT-II nTreg were specifically boosted in their proliferation in PLNs of RelB^DCko^ mice but not in control mice or in the spleen. Thus, autoreactive Tml stimulated by self-antigen-presenting ssmDCs may respond by secreting IL-2 for nTreg proliferation and thereby help to control the peripheral nTreg pool size.

Besides IL-2 nTreg may require a TCR signal for proliferation. nTregs in RelB^DCko^ mice are also expressing higher levels of activation markers PD-1 and HELIOS and the proliferation marker Ki67, indicating a recent encounter of their TCR. Since the functional task of all ssmDC subsets is self-antigen transport from peripheral tissues such as the skin into draining lymph nodes (2) and the frequency of ssmDCs in PLNs is increased it is most likely that the ssmDCs and no other APC subset is responsible for the increased nTreg and Tml frequencies. The data are consistent with recent genetic evidence that expanded DC populations affect T cell homeostasis, increasing Tconv and Treg subsets and favoring a tolerogenic environment (36). Together, there is evidence that nTreg could respond to antigen presented by DCs in the steady state.

Previous data including our own indicate that harmless soluble OVA antigen as provided by a subcutaneously implanted osmotic minipump (12, 13, 15) and cell-associated self-antigens as mimicked by keratinocyte-restricted OVA neo-self-antigen expression (K5-mOVA) (15) induced tolerance by *de novo* converting naive CD4^+^ T cells into Foxp3^+^ iTregs. We identified the Langerin^+^ ssmDCs subsets in PLNs to be responsible for the iTreg conversion while the specific roles of the two Langerin^+^ subsets LCs and CD103^+^ Langerin^+^ dDCs were not separated (15). Here, we found that CD103^+^ Langerin^+^ dDCs are not increased in their frequency in PLNs of RelB^DCko^ mice and do not show an increased rate of iTreg conversion. In contrast, increased frequencies of epidermal LCs and CD103^−^ Langerin^−^ dDCs in PLNs of RelB^DCko^ mice correlated with increased frequencies of activated and proliferating nTregs. In conclusion, these findings may point to a division of labor among ssmDCs. While CD103^+^ Langerin^+^ dDCs mediate iTreg conversion, CD103^−^ Langerin^−^ dDCs may control nTreg maintenance and pool size. The role of LCs in iTreg and nTreg homeostasis remains unclear by the analysis here and previous findings (15).

RelB appears to be uniformly expressed in ssmDCs and thus marks the partial maturation process (semimaturation) of DCs (9). Partial maturation of ssmDCs is required to express CCR7 and enable their lymph node homing (11, 15). The induction of CCR7 expression in DCs seems to be linked to the upregulation of MHC II and costimulatory molecules by transcription factors IRF-4 and IRF-8 (17). RelB expression is upregulated in ssmDCs but is not a specific characteristic of tolerogenic DCs as proposed by others (10) and can exert immunogenic and tolerogenic function in DCs. RelB-deficient mice develop skin lesions similar to atopic dermatitis, pointing to a tolerogenic role for RelB in the skin and may be LCs (37). However, RelB expression and nuclear translocation can be further enhanced in DCs together with other NF-κB/Rel family members upon full maturation induced by pathogens or inflammation (15). Together, it appears that RelB/p52 heterodimers as expressed in ssmDCs may control the functions of tolerogenic DCs (15) or in lymphorganogenesis, whereas formation of RelB/p50 or p65/p50 canonical dimers promotes inflammatory programs in DCs (38, 39). RelB/p50 heterodimers seem to be generally required for production pro-inflammatory cytokines (40).

The RelB^+^ expression in ssmDCs may not be decisive for their tolerogenicity or immunogenicity. Also, iDCs are able to induce T cell anergy or to induce iTregs (4, 5). Critical for Foxp3 induction in naive T cells as a key transcription factor for iTreg induction are TGF-β and retinoic acid that may be provided by DCs or their environment (3). The expression of surface LAP molecules that keep TGF-β trapped in its inactive form (15) until it is released by the DCs for iTreg induction by αvβ8 integrin activity was shown to play a critical role to prevent autoimmunity (41, 42). In contrast, while the conditions of stably Foxp3-expressing nTreg maintenance have been identified as IL-2 and TCR signals, the specific DC subsets and maturation stage as well as the anatomic site of their stimulation have not been clearly dissected. Our data provide evidence that specific ssmDC subsets differ in their roles of iTreg conversion and nTreg maintenance.

In conclusion, our data provide evidence for a role of RelB expression in ssmDCs to control their migration potential of LCs and Langerin^−^ dDCs in PLNs. The increased frequencies of these two DC subsets in PLNs coincide with an increase in nTreg and IL-2^+^ Tml, suggesting a model where the migratory DCs transport and present self-antigens to both T cell subsets. In this concept, Tml cells might represent autoreactive CD4^+^ T cells expressing only low affinity TCRs. As a consequence, stimulation of such T cells by self-antigens presented on ssmDCs is not sufficient to initiate an autoimmune response but promotes the secretion of low levels of IL-2 to stimulate nTreg proliferation, thereby adjusting the homeostatic pool of nTreg.

## Ethics Statement

Animal housing and experimental studies were approved by the local authorities in Würzburg, Jena and New York.

## Author Contributions

AD designed, performed, and analyzed experiments, interpreted the data, and wrote the paper. TS and IE performed and analyzed experiments. NA, MR, and FW helped with the design of experiments, helped to interpret the data, and provided methods and mice. BR provided methods and mice and helped to interpret the data. ML designed experiments, interpreted the data, and wrote the paper.

## Conflict of Interest Statement

The authors declare that the research was conducted in the absence of any commercial or financial relationships that could be construed as a potential conflict of interest.
