# Supplementary material for: RelB+ Steady-State Migratory Dendritic Cells Control the Peripheral Pool of the Natural Foxp3+ Regulatory T Cells
Source: Front Immunol. 2017 Jun 22;8:726. doi: 10.3389/fimmu.2017.00726 (PMC5479892; doi:10.3389/fimmu.2017.00726)
Supplement: Supplementary file 1 [file Presentation_1.PDF]

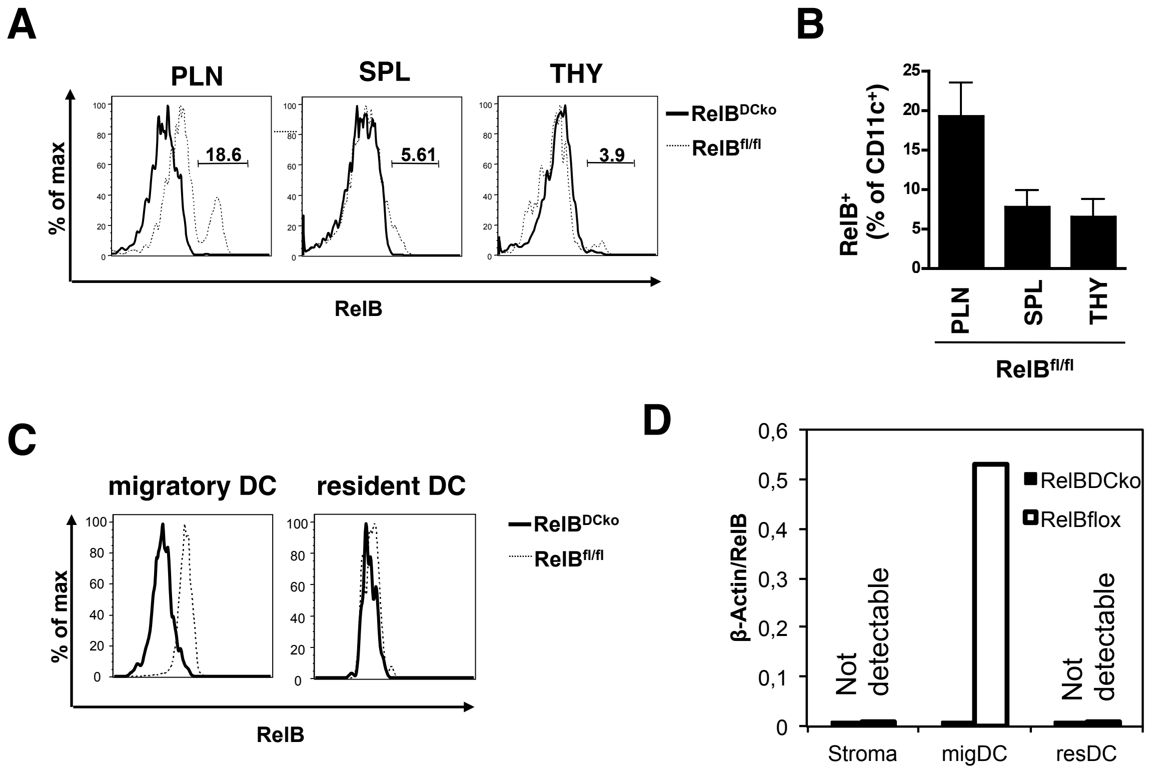

Suppl. Fig. 1: CD11c$^+$ DC of RelB^{DCko} mice fail to express RelB. Flow cytometric analysis of DC from peripheral lymph nodes (PLN), spleen (SPL) and thymi (THY) of RelB$^{fl/fl}$ and RelB^{DCko} mice that were surface stained for CD11c and CD40 and intracellularly stained for RelB. **(A)** Histograms display RelB expression of CD11c$^+$ DC in lymphoid organs. Numbers indicate the percentages of RelB$^+$ cells within CD11c$^+$ DC of RelB$^{fl/fl}$ mice. **(B)** Percentages of RelB$^+$ cells among CD11c$^+$ DC in the lymphoid organs of RelB$^{fl/fl}$ mice (n = 4). Data represent the mean values + SD. **(C)** Histograms show RelB expression of steady state migratory CD40^{hi} CD11c$^+$ DC and resident CD40^{low} CD11c$^+$ DC in PLN of RelB$^{fl/fl}$ and RelB^{DCko} mice. (D) qRT-OCR analysis of RIB expression normalized to β-actin for the indicated cell types.

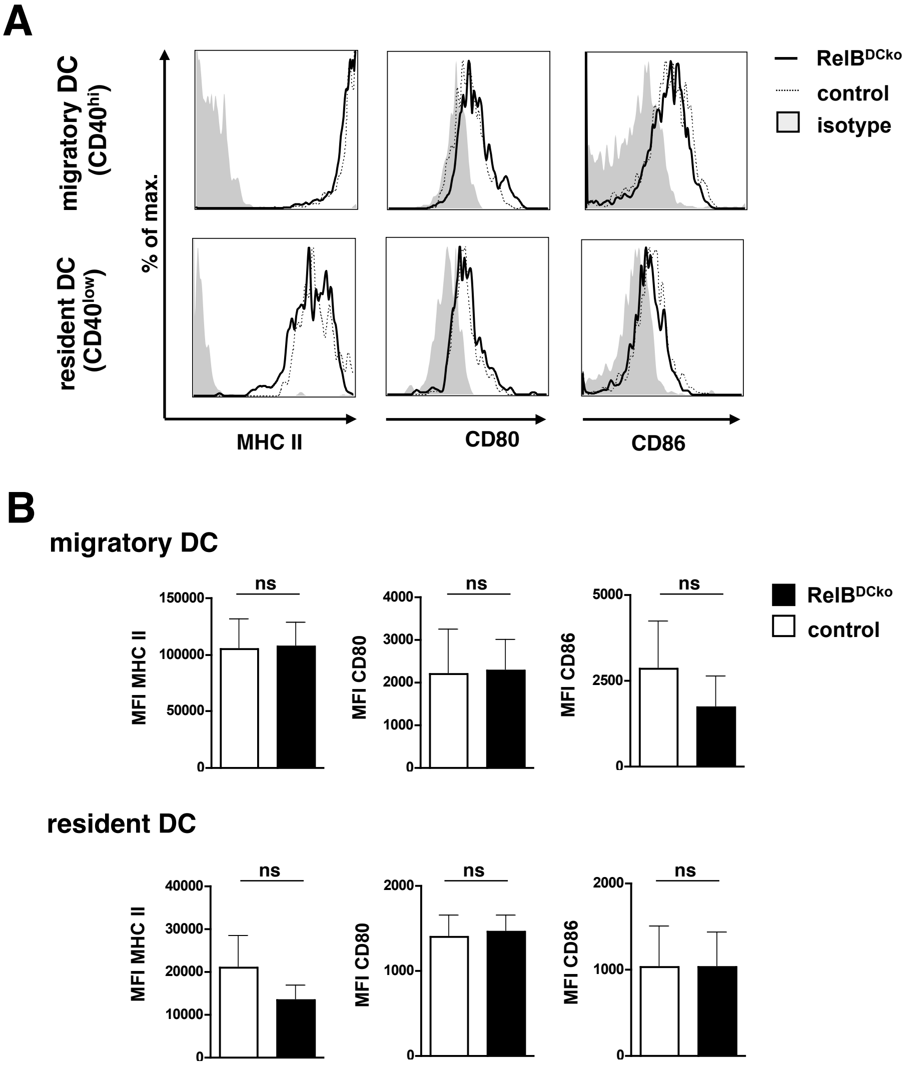

Suppl. Fig. 2: RelB^{DCko} mice show no difference in the indicated surface marker expression compared to control mice. (A) Expression analysis of MHC II, CD80 and CD86 on migratory CD40^{hi} DC and resident CD40^{low} DC in PLN of RelB^{DCko} and control mice. Dotted lines represent control DC, solid lines show RelB^{DCko} DC and gray histograms isotype controls. Data shown are representative of at least three independent experiments with similar results. **(B)** Geometric mean fluorescence intensities (MFI) of MHC II, CD80 and CD86 on migratory CD40^{hi} DC and resident CD40^{low} DC in PLN of control mice ($n \geq 4$) and RelB^{DCko} mice ($n \geq 4$). Graphs show mean values + SD. Statistical analyses were performed using the Mann-Whitney Test: ns - not significant, * $p < 0.05$.

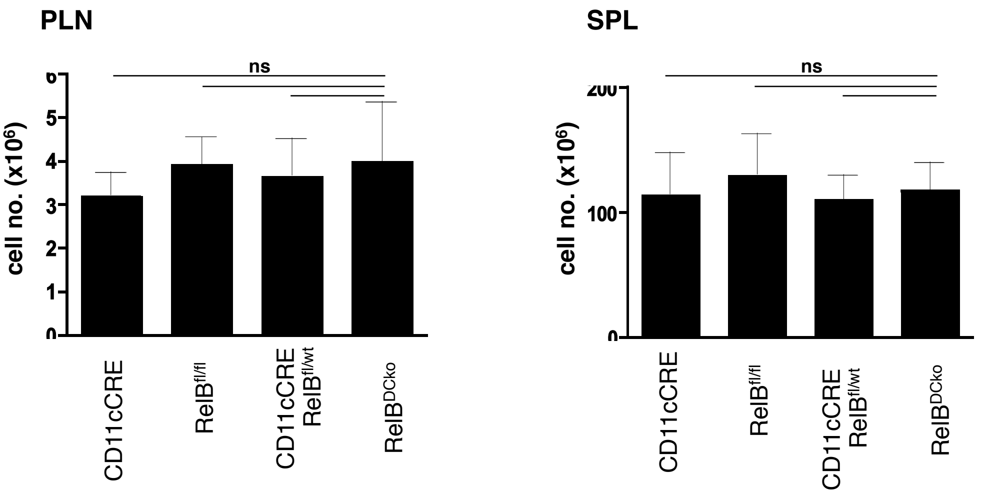

Suppl. Fig. 3: RelB^{DCko} mice show no difference in the cellularities of peripheral lymphoid organs compared to control mice. Cell suspensions of PLN and SPL from CD11cCRE (n = 3), RelB$^{fl/fl}$ (n = 5), CD11cCRE x RelB$^{fl/wt}$ (n = 7) and RelB^{DCko} (n = 11) mice were analyzed regarding their cell number. Data represent the mean values + SD. Statistical analysis was performed using Mann-Whitney Test: ns - not significant.

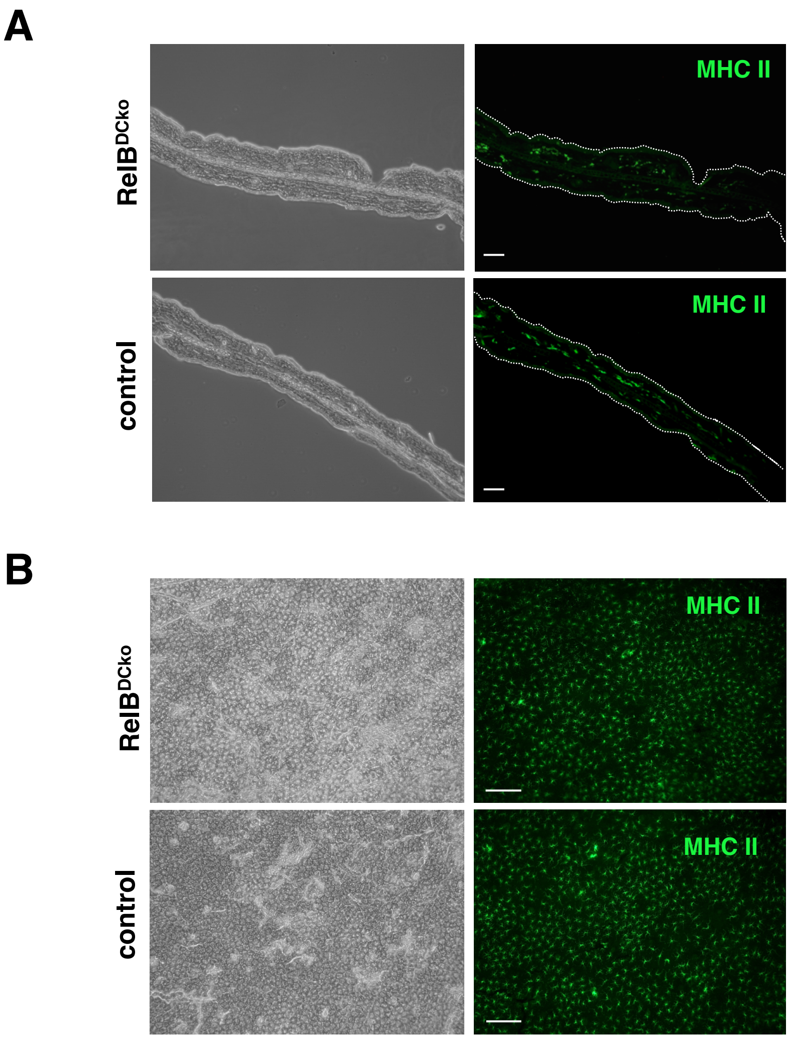

Suppl. Fig. 4: Normal distribution of DC in the skin of RelB^{DCko} mice. (A) Cryostat sections of ear skin and **(B)** epidermal sheets were fixed in acetone, labeled with anti-MHC II in combination with anti-Rat IgG2a-AlexaFluor488 and analyzed by microscopy. Representative phase contrast micrographs (left) and fluorescence micrographs (right) of ear skin and epidermal sheets from RelB^{DCko} and control mice as well as compiled statistical data for counted MHC II^{+} cells within epidermal sheets are shown. Scale bars represent 200 µm. Two animals per genotype from two independent experiment were analyzed with similar resilts.

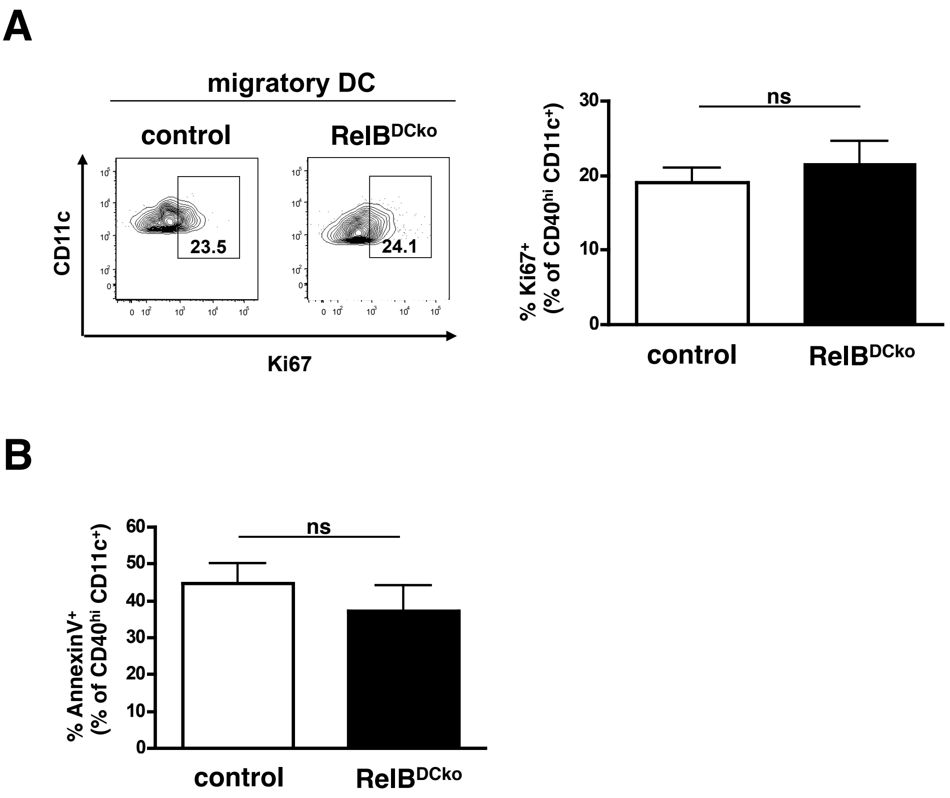

Suppl. Fig. 5: Steady state migratory DC in peripheral lymph nodes of RelB^{DCko} mice display no alterations in proliferation and apoptosis compared to control mice. (A, B) Cells from PLN were stained for CD11c, CD40, PDCA-1 and either Ki67 or AnnexinV. Percentages of Ki67^{+} or AnnexinV^{+} cells among CD40^{hi} CD11c^{+} pregtaed on PDCA-1^{-} CD11c^{+} cells in PLN of control mice (n = 4) and RelB^{DCko} mice (n = 4) are shown. Data represent the mean values + SD from at least two individual experiments. Statistical analyses were performed using Mann-Whitney Test: ns - not significant.

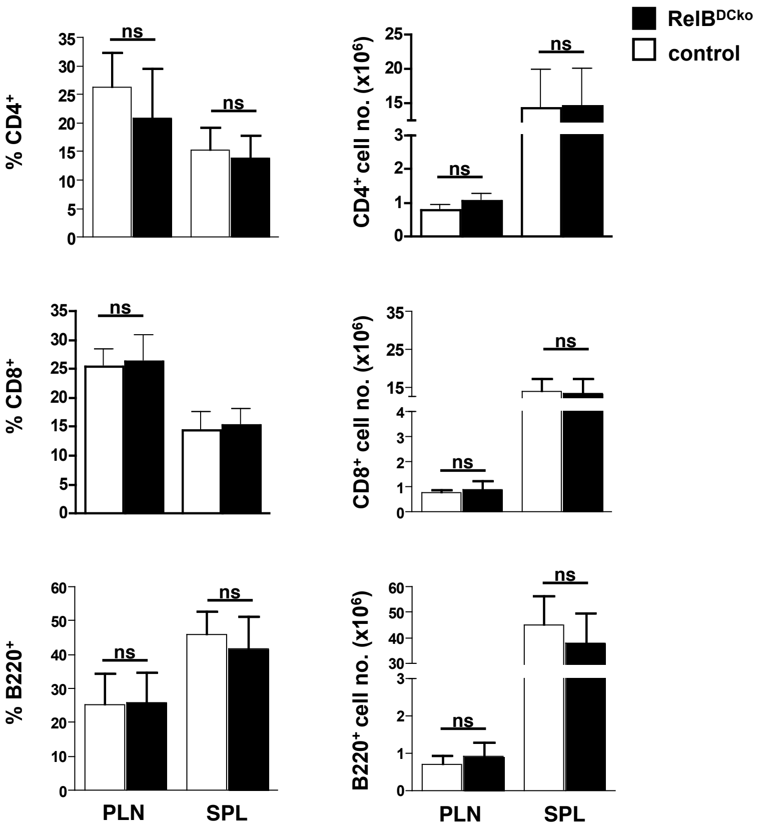

Suppl. Fig. 6: RelB^{DCko} mice display an unaltered frequency and total cell number of T and B cells in peripheral lymphoid organs.
Percentages and absolute cell numbers of CD4^{+} T cells, CD8^{+} T cells and B220^{+} B cells among total living cells in PLN and SPL of control mice (n = 7) and RelB^{DCko} mice (n = 5). Data represent the mean values + SD. Statistical analyses were performed using Mann-Whitney Test: ns - not significant.

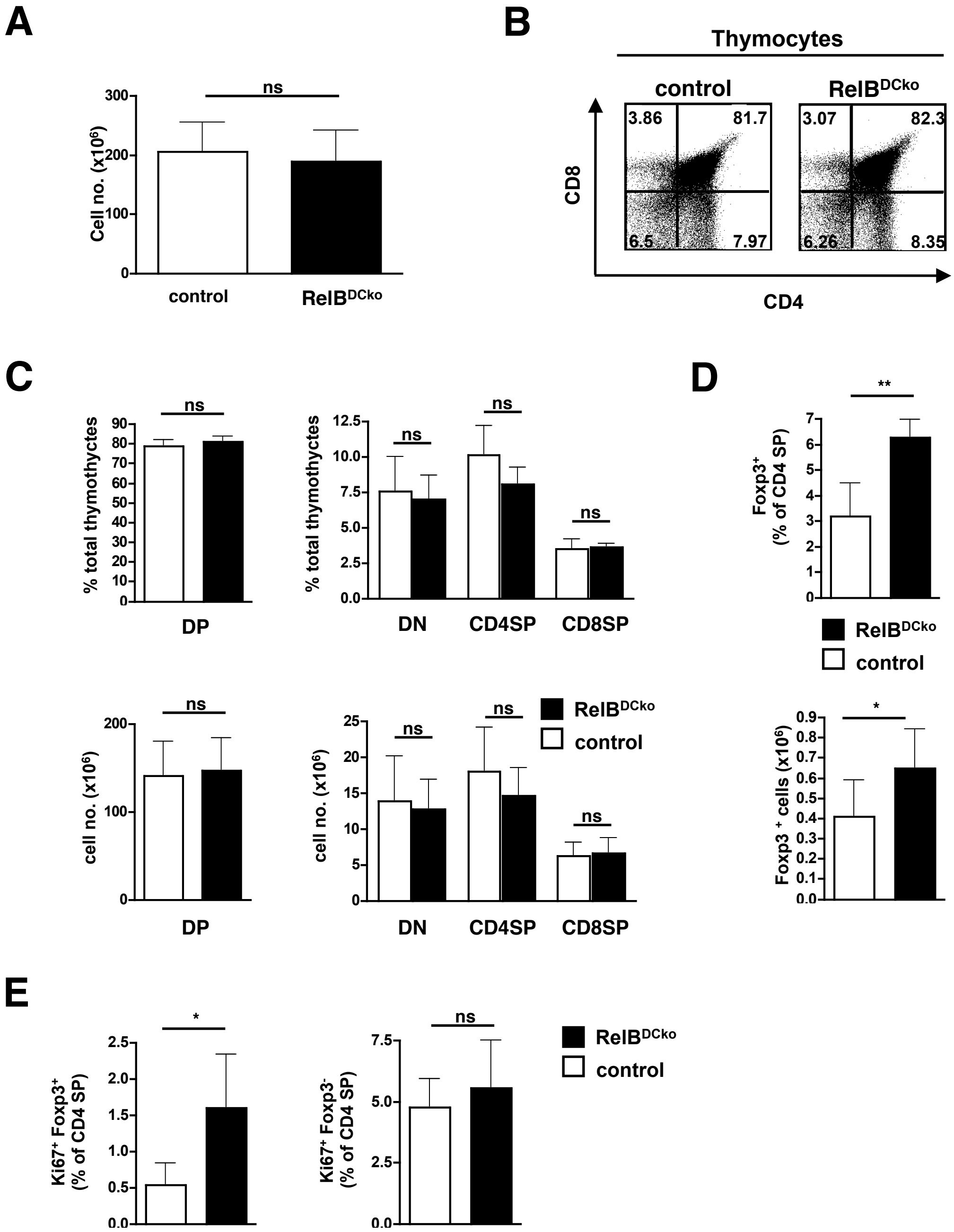

Suppl. Fig. 7: Increased frequency of Foxp3$^+$ T$_{regs}$ in the thymus of RelB^{DCko} mice. **(A)** Cellularities of thymi from control and RelB^{DCko} mice are displayed. **(B)** Representative flow cytometry analyses of CD4 and CD8 expression among thymocytes are shown. Numbers indicate the percentages of gated thymocyte populations among total thymocytes of control and RelB^{DCko} mice. **(C)** Percentages and absolute numbers of CD4$^+$ CD8$^+$ double positive thymocytes (DP), CD4$^-$ CD8$^-$ double negative thymocytes (DN), CD4$^+$ single positive thymocytes (CD4SP) and CD8$^+$ single positive thymocytes (CD8SP) within total thymocytes in thymi of control mice (n = 8) and RelB^{DCko} mice (n = 6). **(D)** Frequencies and absolute numbers of CD4$^+$ Foxp3$^+$ T$_{regs}$ within CD4SP cells in thymi of control mice (n = 6) and RelB^{DCko} mice (n = 4). **(E)** Percentages of proliferating Ki67$^+$ Foxp3$^+$ T$_{regs}$ and Ki67$^+$ Foxp3$^-$ T$_{conv}$ among CD4SP cells in thymi of control mice (n = 6) and RelB^{DCko} mice (n = 4). **(A, C- E)** Shown are mean values + SD. Statistical analyses were performed using Mann-Whitney Test: ns - not significant; * $p < 0,05$; ** $p < 0,01$.
